# The role of reactive oxygen intermediates in the intracellular fate of *Leptospira interrogans* in the macrophages of different hosts

**DOI:** 10.1371/journal.pone.0178618

**Published:** 2017-06-02

**Authors:** Shijun Li, Peili Li, Lei Zhang, Weilin Hu, Ming Wang, Ying Liu, Guangpeng Tang, Dingming Wang, Bijun Zhou, Jie Yan

**Affiliations:** 1 Institute of Communicable Disease Control and Prevention, Guizhou Provincial Center for Disease Control and Prevention, Guiyang, Guizhou, P.R. China; 2 College of Animal Science, Guizhou University, Huaxi District, Guiyang, Guizhou, P.R. China; 3 Zhejiang Provincial Center for Disease Control and Prevention, Hangzhou, Zhejiang, P.R. China; 4 Department of Medical Microbiology and Parasitology, College of Medicine, Zhejiang University, Hangzhou, P.R. China; Cornell University, UNITED STATES

## Abstract

**Background:**

Pathogenic species of *Leptospira* cause leptospirosis, a global zoonotic disease. Our previous work showed that leptospires survive and replicate in human macrophages but are killed in murine macrophages. However, the mechanism responsible for the different intracellular fates of leptospires within the macrophages of different hosts remains unclear.

**Results:**

The present study demonstrates that infection with *Leptospira interrogans* caused significant up-regulation of reactive oxygen species (ROS) and superoxide in J774A.1 cells but did so to a lesser extent in THP-1 cells. The up-regulation of ROS and superoxide was significantly inhibited by the NADPH oxidase inhibitor apocynin. The damaged leptospires and remnants of leptospires within membrane-bound vacuoles were significantly inhibited by apocynin in J774A.1 cells but were less inhibited in THP-1 cells. In addition, apocynin significantly prevented damage to leptospires and the co-localization of *L*. *interrogans* with lysosomes in J774A.1 cells but did so to a lesser extent in THP-1 cells. Furthermore, the relative fluorescence intensity levels of intracellular leptospires and the viability of the intracellular leptospires increased in apocynin pretreated J774A.1 and THP-1 cells after 2 h of infection.

**Conclusions:**

The present study, based on our previous findings, further demonstrated that ROS contributed substantially to the bactericidal ability of mouse macrophages to kill intracellular leptospires. However, ROS did not contribute as much in human macrophages, which partially explains the different intracellular fates of *L*. *interrogans* in human and mouse macrophages.

## Introduction

Pathogenic *Leptospira* spp. are the causative agents of leptospirosis [1, 2], which is the most widespread zoonotic disease in the world [3]. Leptospirosis has emerged as a major public health burden in urban slums, with a recent estimate of 1 million cases per year [4–6]. It occurs in highly populated, poor urban centers where flooding frequently occurs [7]. Rodents constitute the main reservoir of leptospires, and they asymptomatically excrete the bacteria in their urine throughout their lifetime. Humans can be infected through contact with contaminated water and soil [1, 7]. Pathogenic leptospires are able to infect humans and many domestic and wild animals, and they then survive and grow in host tissues by escaping natural defense mechanisms [3]. Human leptospirosis has many different symptoms, varying from a flu-like syndrome to multiorgan failure that leads to death [7]. Pulmonary diffuse hemorrhage, a serious clinical form of leptospirosis, is fatal in approximately 40–50% of patients [8, 9]. In contrast, maintenance hosts are typically asymptomatic, and *Leptospira* evades the immune response to colonize renal tubules from which they are shed in urine [10]. However, the reason why the outcomes of *Leptospira* infection differ in humans and reservoir hosts remains unknown.

Phagocytes play a critical role in innate immunity against invading pathogens. Mononuclear macrophages and neutrophils have been shown to phagocytose leptospires, but only the former can kill the phagocytosed intracellular leptospires [11, 12], indicating that mononuclear macrophages are much more important than neutrophils in the defense mechanisms against leptospiral infection. Our previous study showed that *L*. *interrogans* survives and replicates within human macrophages but not within murine macrophages [13], which suggested that the bactericidal mechanisms of the human and murine macrophages are important for determining the intracellular fate of leptospires, but the bactericidal mechanisms need to be further studied.

The generation of reactive oxygen intermediates (ROIs) by macrophages occurs during the phagocytosis of many bacteria, fungi, and protozoa [14]. Resident macrophages have some capacity to undergo a respiratory burst, but immunologic activation of macrophages substantially augments this process [14]. The antimicrobial activity of ROIs has been demonstrated by a variety of approaches and has been most strongly implicated in host defenses against intracellular pathogens such as *Listeria monocytogenes*, *Mycobacterium avium* and *Francisella tularensis* [15–18]. Infection with *L*. *interrogans* has been shown to stimulate the production of high levels of reactive oxygen intermediates (ROIs) in rat Kupffer cells in liver tissues [19], and infection with *Leptospira interrogans* caused a rapid increase in ROIs in mouse and human macrophages [12]. Thus, we hypothesized that ROIs participate in the bactericidal mechanisms of human and mouse macrophages. Therefore, in the present study, human monocytes (THP-1 cell line) and murine mononuclear macrophages (J774A.1 cell line) were used to characterize ROI changes due to infection with *L*. *interrogans* and to determine the role of ROIs in the intracellular fate of *L*. *interrogans* in the macrophages of different hosts.

## Material and methods

### Leptospiral strain and cultivation

The *L*. *interrogans* serovar Lai strain Lai used in this study was provided by the Chinese Center for Disease Control and Prevention (Beijing, China), and it was cultivated in Ellinghausen–McCullough–Johnson–Harris (EMJH) liquid medium at 28°C [13, 20, 21]. To maintain virulence, the strain was intraperitoneally passaged in specific pathogen-free Dunkin–Hartley ICO:DH (Poc) guinea pigs (10–12 d old, each weighing approximately 120 g) before use, according to the description by Merien et al. and Viriyakosol et al [8, 21]. Animal protocols were approved by the Animal Ethics Review Committee of the Guizhou Provincial Center for Disease Control and Prevention. Low-passage-number isolate were used in the infection experiment.

### Cell lines and culture conditions

The murine monocyte macrophage-like cell line (J774A.1) and the human monocytic cell line (THP-1) were from the American Type Culture Collection (ATCC; Rockville, MD, USA). Both J774A.1 and THP-1 cells were cultured using RPMI-1640 medium (Gibco Laboratories; NY, USA) containing 10% heat-inactivated fetal calf serum (FCS; Gibco), 100 U/ml penicillin and 100 mg/ml streptomycin (Sigma Chemical Co.; St Louis, MO, USA) at 37°C in an atmosphere of 5% CO_2_. Before infection, THP-1 cells were treated with 10 ng/ml PMA for 24 h to differentiate them into macrophages [13, 22].

### Detection of intracellular ROS

The ROS levels in THP-1 or J774A.1 cells infected with *L*. *interrogans* strain Lai were detected using an ROS specific fluorescent dye, dichlorofluorescein diacetate (DCFH-DA) (Sigma) [12]. Briefly, freshly cultured *L*. *interrogans* strain Lai cells were harvested by centrifugation (16,000 g at 15°C for 15 min). THP-1 or J774A.1 cells (1 x 10^5^ per well) were seeded into 12-well culture plates (Corning, USA) containing a 12 x 12 mm coverslip in each well for incubation overnight at 37°C. The coverslips with THP-1 or J774A.1 cell monolayers were washed thoroughly with PBS and then infected with the harvested *L*. *interrogans* at a multiplicity of infection (MOI) of 100 (100 leptospires per host cell) for co-incubation at 37°C for 2, 4, 12 or 24 h [13, 23]. After washing with PBS, the infected macrophages were incubated in antibiotic-free 2.5% FCS RPMI-1640 medium containing 5 mM DCFH-DA for 30 min at 37°C. The fluorescence intensity, which reflects intracellular ROS levels, was detected using a laser confocal microscope (Olympus, Tokyo, Japan) with 488 nm excitation and 530 nm emission wavelengths. Images were captured with a Fluoview FV1000 camera (Olympus, Tokyo, Japan). Final image processing was performed using the FluoView viewer (version 1.7.a; Olympus). For inhibitory tests, cells were pretreated with apocynin (100 μmol/L) (Sigma-Aldrich, St. Louis, Mo.) to inhibit NADPH oxidase activity [24, 25] at a final concentration of 0.5 μmol/L for 1 h at 37°C, and the subsequent experimental steps were the same as described above.

### Detection of superoxide

Superoxide was measured using a superoxide assay kit (Beyotime, China) as previously described [26]. Briefly, THP-1 or J774A.1 cells (1 x 10^5^ per well) were seeded into 12-well culture plates (Corning, USA) for incubation overnight at 37°C. The cell monolayers were washed thoroughly with PBS and then infected with harvested *L*. *interrogans* strain Lai at a MOI of 100 for co-incubation at 37°C for 2, 4, 12 or 24 h as described above. Because superoxide can deoxidize WST-1 and produce a soluble orange formazan [26, 27], the optical density of each microplate was measured at 450 nm using a Bio-RAD microplate reader (Bio-Rad, Hercules, CA). For inhibitory tests, cells were pretreated with apocynin (100 μmol/L) (Sigma-Aldrich, St. Louis, Mo.) to inhibit NADPH oxidase activity at a final concentration of 0.5 μmol/L for 1 h at 37°C, and the subsequent experimental steps were the same as described above.

### Determination of the distribution of intracellular leptospires

Transmission electron microscopy (TEM) was used to observe the distribution of leptospires in J774A.1 and THP-1 cells as previously described [13, 28, 29]. Briefly, after infection with *L*. *interrogans* strain Lai at an MOI of 100 at 37°C for 1 h, the cells were washed three times with sterile PBS, and then 50 μg/mL of gentamicin (Sigma) was added to kill the remaining extracellular leptospires. After co-incubation for 4, 12, or 24 h, the cells were collected with a cell scratcher and centrifugation (1500 g, 10 min, 4°C) and were then fixed with 2.5% formaldehyde, post-fixed with 1% osmium tetroxide, dehydrated, embedded in Epon (Sigma), cut with a diamond knife, and collected on 100–150 mesh nickel grids (Plano; Wechsler, Germany). The sections were examined using transmission electron microscopy (TEM) (Olympus, Tokyo, Japan). For inhibitory tests, cell monolayers that were pretreated with apocynin (100 μmol/L) as described above were used as a control to determine the potential influence of ROS on the distribution of intracellular leptospires.

### Determination of intracellular leptospires co-localization with lysosomes

The co-localization of intracellular leptospires with the late-endosomal/lysosomal marker LAMP-1 was determined using immunofluorescence staining as previously described [30].

Briefly, coverslips with THP-1 or J774A.1 cell monolayers were infected with *L*. *interrogans* strains Lai at an MOI of 100. After incubation for 4, 12 or 24 h, the cells were washed with sterile PBS, fixed with 4% paraformaldehyde for 20 min and permeabilized with PBS containing 3% non-fat milk and 0.05% saponin (Sigma). The cell samples were labeled for 30 min with 1:200 diluted rabbit antiserum against *L*. *interrogans* strain Lai, mouse anti-human LAMP-1 monoclonal antibody (eBioscience) or rat anti-mouse LAMP-1 monoclonal antibody (eBioscience). After three washes with sterile PBS, the samples were labeled with 1:400 diluted Alexa Fluor 568-conjugated goat anti-rabbit F(ab’)2, Alexa Fluor 488-conjugated goat anti-mouse F(ab’)2 (Invitrogen) or Alexa Fluor 488-conjugated goat anti-rat F(ab’)2 (Invitrogen) for 30 min. The samples were then stained with 1 μg ml^-1^ DAPI (Invitrogen) for 5 min and examined under a laser scanning confocal microscope (Olympus, Tokyo, Japan). Images were captured with a Fluoview FV1000 camera (Olympus, Tokyo, Japan). Final image processing was performed using the FluoView viewer (version 1.7.a; Olympus). The percentage of cells in which lysosome co-localized with leptospire was counted. A totall of 100 cells were counted to caculate the percentage of co-localization. Cell monolayers that were pretreated with apocynin (100 μmol/L) as described above were used as a control to determine the potential influence of ROS on the co-localization of intracellular leptospires with the late-endosomal/lysosomal marker LAMP-1.

### Quantification of intracellular leptospires

Immunofluorescence staining was performed to quantify the intracellular leptospires in J774A.1 and THP-1 cells as previously described [30]. Briefly, coverslips with THP-1 or J774A.1 cell monolayers were infected with *L*. *interrogans* strains Lai at an MOI of 100 and were incubated for 4, 12 or 24 h. After three washes with sterile PBS, the cell slips were fixed with 4% paraformaldehyde for 20 min and permeabilized with PBS containing 3% non-fat milk and 0.05% saponin (Sigma). The cell samples were incubated with 1:200 diluted rabbit antiserum against *L*. *interrogans* strain Lai for 30 min. After three washes with PBS, the samples were incubated for 30 min with 1:400 diluted Alexa Fluor 568-conjugated goat anti-rabbit F(ab’)2. Finally, the samples were stained with 1 μg ml^-1^ DAPI (Invitrogen) for 5 min and examined under a laser scanning confocal microscope (Olympus, Tokyo, Japan). Images were captured with a Fluoview FV1000 camera (Olympus, Tokyo, Japan). Final image processing was performed using the FluoView viewer (version 1.7.a; Olympus). The fluorescence intensity of intracellular leptospires in 100 infected cells was counted. In the confocal microscopic detection, cell monolayers that were pretreated with apocynin (100 μmol/L) as described above were used as a control to determine the potential influence of ROS on the amount of intracellular leptospires.

### Viability assessment of intracellular leptospires

J774A.1 and THP-1 cells were infected with *L*. *interrogans* strain Lai at an MOI of 100 and were then treated with gentamicin (50 μg/mL) to kill the remaining extracellular leptospires. After incubation for 2, 4, 12 or 24 h, the cells were lysed with 0.05% sodium deoxycholate (Sigma) in sterile PBS. The lysate cell debris was removed by centrifugation at 1500 g for 2 min, and the supernatants were centrifuged at 15,000 g for 10 min at 15°C to precipitate the leptospires. Viability was determined using a Bacterial Viability Kit (Invitrogen) according to the manufacturer’s instructions. Briefly, the collected leptospires were stained with SYTO 9 and propidium iodide (PI) for 15 min at room temperature. The live/dead leptospires in the mixture were determined by flow cytometry (FACSCalibur flow cytometer, BD Biosciences, NJ, USA)[13, 31, 32], and the percentage of live leptospires was calculated. Additionally, the mobility of the leptospires was observed under a dark-field microscope (Olympus, Tokyo, Japan). For the inhibitory tests, cell monolayers that were pretreated with apocynin (100 μmol/L) as described above were used as a control to determine the potential influence of ROS on the viability of *L*. *interrogans* in mouse and human macrophages.

### Statistical analysis

The data are presented as the mean ± SD (standard deviation), and the *t*-test was used to determine significant differences. Statistical significance was defined as *P* <0.05.

## Results

### Increase in ROS levels in leptospire-infected macrophages

**Fig 1:**
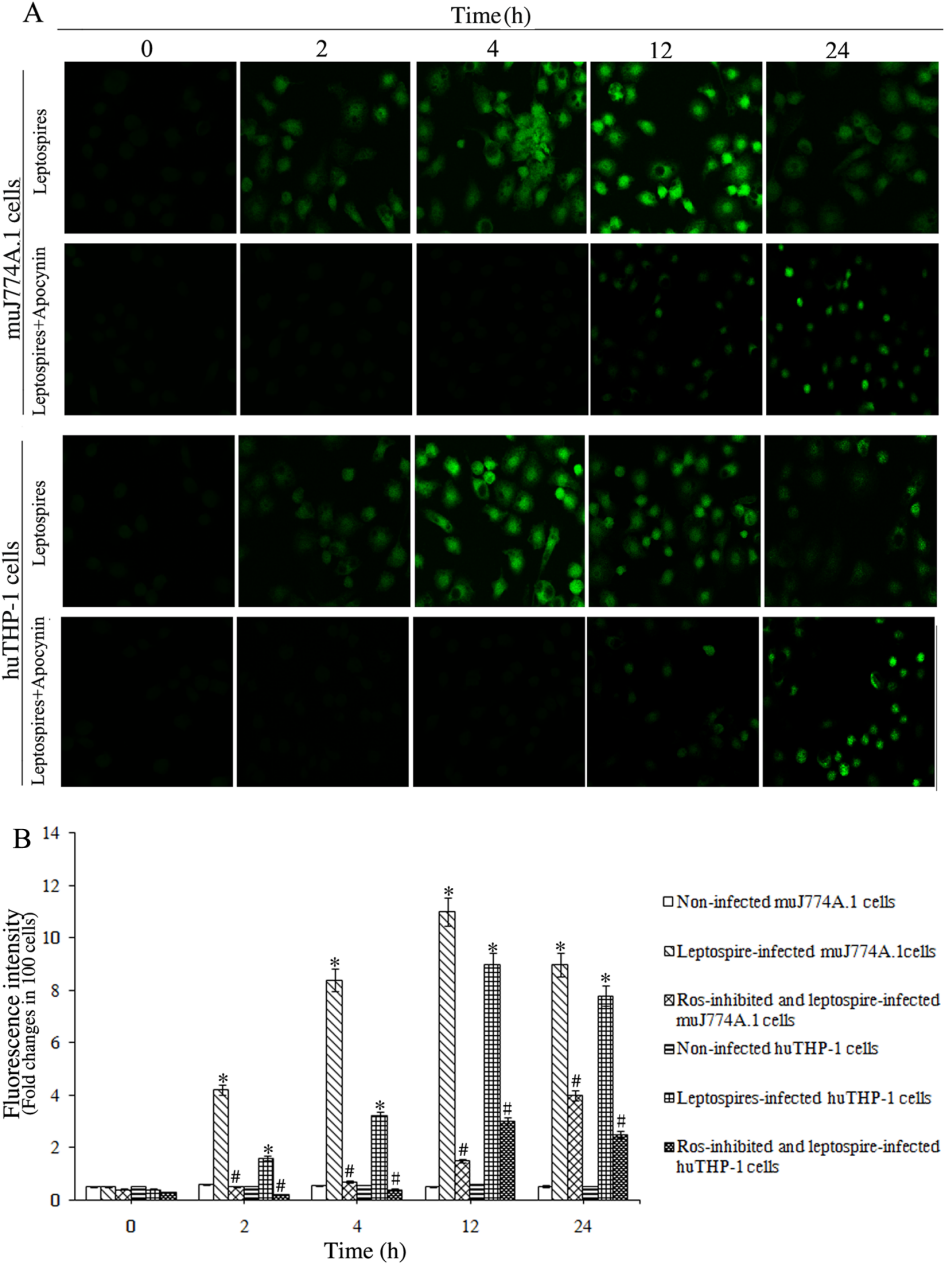
Intracellular ROS levels of human and murine macrophages infected with *L*. *interrogans*. Cells were infected with *L*. *interrogans* strain Lai at an MOI of 100 for different time periods and stained with DCFH-DA, Apocynin was used to inhibit the production of ROS. The 0 h time point indicates the ROS levels in the cells before infection. A. Intracellular ROS levels in J774A.1 or THP-1 cells infected with *L*. *interrogans* strain Lai for the indicated times. B. Fluorescence intensity reflecting the ROS levels in leptospire-infected cells for the indicated times. Statistical data were from the experiments shown in (A). Bars indicate the mean ± SD of three independent experiments. One hundred cells in each experiment were analyzed to quantify each fluorescence signal intensity value. The 0 h time point shows the ROS levels in the cells before infection. *P < 0.05 versus the ROS levels in the cells before infection. ^#^*P* < 0.05 versus the ROS levels in the cells without apocynin inhibition.

The ROS-specific fluorescent staining assays confirmed that ROS levels were significantly increased in J774A.1 and THP-1 cells 2 h after infection with *L*. *interrogans* strain Lai, and the maximal intracellular ROS levels appeared after 12 h of infection, with significantly lower levels in THP-1 cells compared to J774A.1 cells (*P*<0.05, Fig 1A and 1B). The leptospire-induced ROS increase in the J774A.1 and THP-1 cells was significantly inhibited by apocynin, an NADPH oxidase inhibitor, at the early stages of infection (*P*<0.05, Fig 1A and 1B).

### Increase in superoxide levels in leptospire-infected macrophages

**Fig 2:**
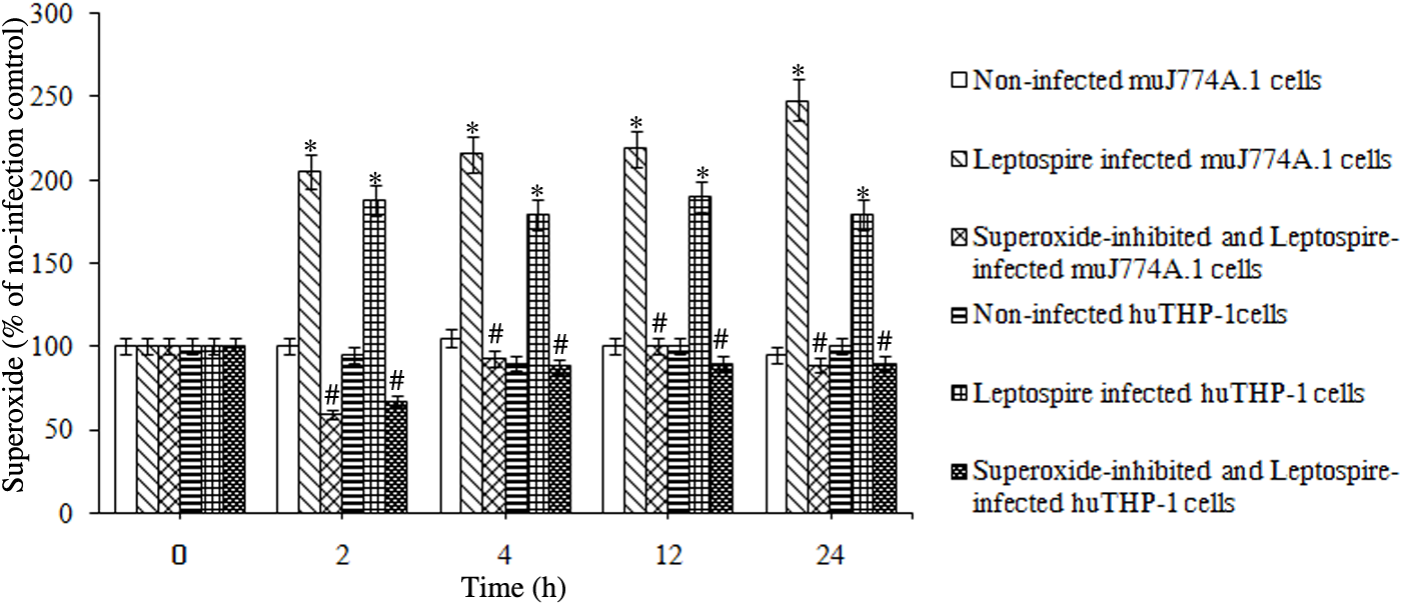
Superoxide levels of human and murine macrophages infected with *L*. *interrogans*. Cells were infected with *L*. *interrogans* strain Lai at an MOI of 100 for different time periods. The superoxide levels of the cells were detected using a superoxide assay kit. Apocynin was used to inhibit the production of superoxide production. The 0 h time point indicates the superoxide levels in the cells before infection. Bars indicate the mean ± SD of three independent experiments. *P < 0.05 versus the superoxide levels in the cells before infection. ^#^*P* < 0.05 versus the superoxide levels in the cells without apocynin inhibition for the indicated times.

Superoxide detection results obtained using a superoxide assay kit confirmed that the superoxide levels were significantly increased in J774A.1 and THP-1 cells after infection with *L*. *interrogans* strain Lai for 4 h, 12 h and 24 h, with a slower increase of ROS in THP-1 cells compared to J7774A.1 cells (*P*<0.05, Fig 2). The NADPH inhibitor apocynin significantly reduced the *Leptospira*-induced superoxide production in both J774A.1 and THP-1 cells (*P*<0.05, Fig 2).

### Distribution of *L*. *interrogans* strain Lai in macrophages

**Fig 3:**
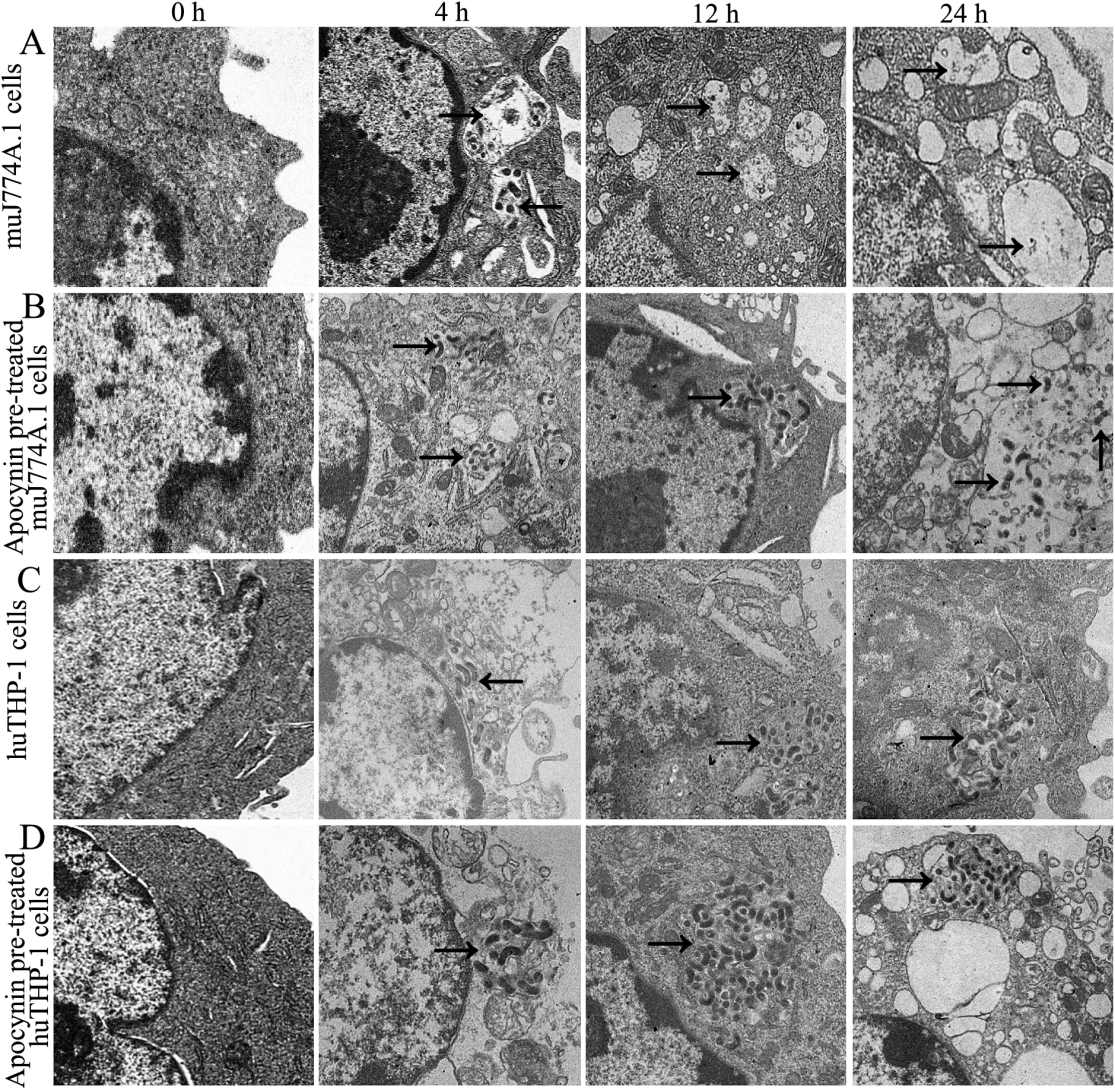
Intracellular localization of *L*. *interrogans* in human and murine macrophages. Cells were infected with *L*. *interrogans* strain Lai at an MOI of 100 for different time periods, the extracellular leptospires were killed by gentamicin, and the intracellular leptospires were observed under TEM. Apocynin was used to inhibit the production of ROS. A. Leptospires (indicated by arrows) in a phagocytic vesicle in a J774A.1 cell. B. Leptospires (indicated by arrows) in apocynin pretreated J774A.1 cell, showing the absence of a surrounding vesicular membrane. C. Leptospires (indicated by arrows) in the cytosol of a THP-1 cell, showing the absence of a surrounding vesicular membrane. D Leptospires (indicated by arrows) in the cytosol of apocynin pretreated THP-1 cell, outside of phagosomes. Bars = 2.0 μm.

The results of the intracellular localization of leptospires with TEM demonstrated that the time-dependent appearance of damaged spiral shape of leptospires and the remnants of damaged leptospires in vacuoles decreased significantly in NADPH oxidase inhibitor apocynin pretreated J774A.1 cells compared to infected J774A.1 cells without apocynin pretreatment (Fig 3). In addition, leptospires that did not have a surrounding vesicular membrane were observed in the cytosol of apocynin pretreated J774A.1 cells, and the typical spiral shape and number of leptospires increased in a time-dependent manner; however, fewer of these changes were observed in apocynin pretreated THP-1 cells (Fig 3).

### Co-localization of *L*. *interrogans* strain Lai with lysosomes in macrophages

**Fig 4:**
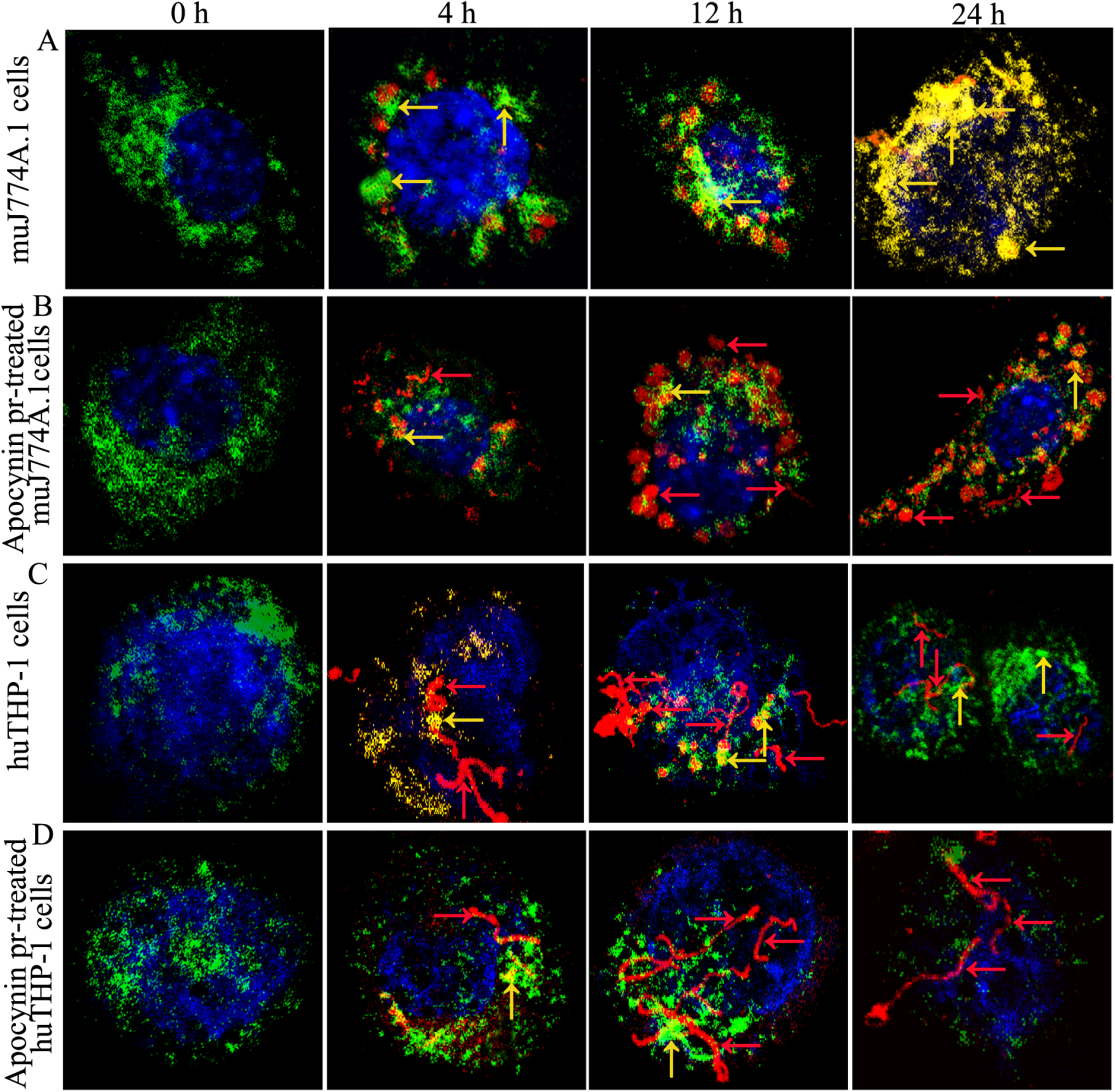
Co-localization of *L*. *interrogans* and lysosomes in human and murine macrophages. Cells were infected with *L*. *interrogans* strain Lai at an MOI of 100 for different time periods, the extracellular leptospires were killed by gentamicin, and the co-localization of leptospires with lysosomes in macrophages was determined by confocal microscopy. Apocynin was used to inhibit the production of ROS. Red indicates intracellular leptospires, green indicates the lysosomal marker LAMP-1, and blue indicates nuclei. Yellow shows the co-localization of leptospires (red) with LAMP-1 (green). A. Representative image from a leptospire-infected J774A.1 cell. B. Representative image from a leptospire-infected J774A.1 cell pretreated with NADPH oxidase inhibitor apocynin. C. Representative image from a leptospire-infected THP-1 cell. D. Representative image from a leptospire-infected THP-1 cell pretreated with NADPH oxidase inhibitor apocynin. Red arrows indicate leptospires and yellow arrows indicate co-localization of leptospires with LAMP-1. Bars = 5 μm.

**Fig 5:**
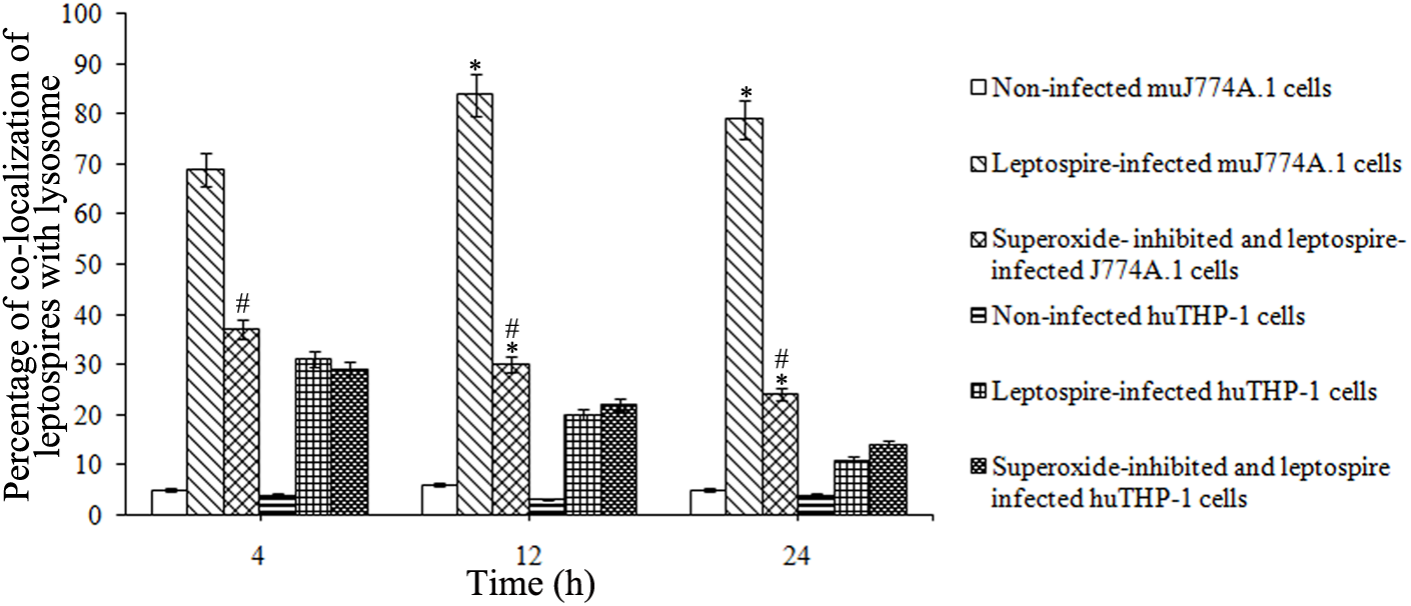
Percentages of *L*. *interrogans* that co-localize with lysosomes in macrophages. J774A.1 and THP-1 cells were infected with *L*. *interrogans* strain Lai at an MOI of 100 for different time periods. The extracellular leptospires were killed by gentamicin, and the percentages of co-localizing leptospires and lysosomes in macrophages were determined by confocal microscopy. Apocynin was used to inhibit the production of ROS. A minimum of 100 infected cells were measured in each experiment, and the data are expressed as the mean ± SD for three independent experiments. *P<0.05 versus 4 h of infection. ^#^*P* < 0.05 versus the cells without apocynin inhibition for the indicated times.

Confocal microscopic images revealed clear co-localization of intracellular leptospiral strain Lai with lysosomes in the J774A.1 cells but not in THP-1 cells (Fig 4). When the cells were infected for 4 h, only small part of the leptospires in THP-1 cells were co-localized with LAMP-1, while most of the leptospires in J774A.1 cells co-localized with LAMP-1(Fig 4). Furthermore, at longer incubation times, the percentages of co-localizing intracellular leptospires in THP-1 cells gradually decreased, whereas those in J774A.1 cells increased progressively (Fig 4 and Fig 5). In addition, confocal microscopic images also revealed a clear inhibitory effect of NADPH oxidase inhibitor apocynin on the co-localization of intracellular leptospiral strain Lai with lysosomes in J774A.1 cells, but this inhibition was lower in THP-1 cells (Fig 4). For instance, the time-dependent appearance of *L*. *interrogans* strain Lai co-localized with lysosomes significantly decreased in NADPH oxidase inhibitor apocynin pretreated J774A.1 cells after infection for 4, 12 and 24 h, but less significant changes in the co-localization of *L*. *interrogans* strain Lai with lysosomes were observed in apocynin pretreated THP-1 cells compared to THP-1 cells without apocynin pretreatment (Fig 4 and Fig 5).

### Quantification of *L*. *interrogans* strain Lai in macrophages

**Fig 6:**
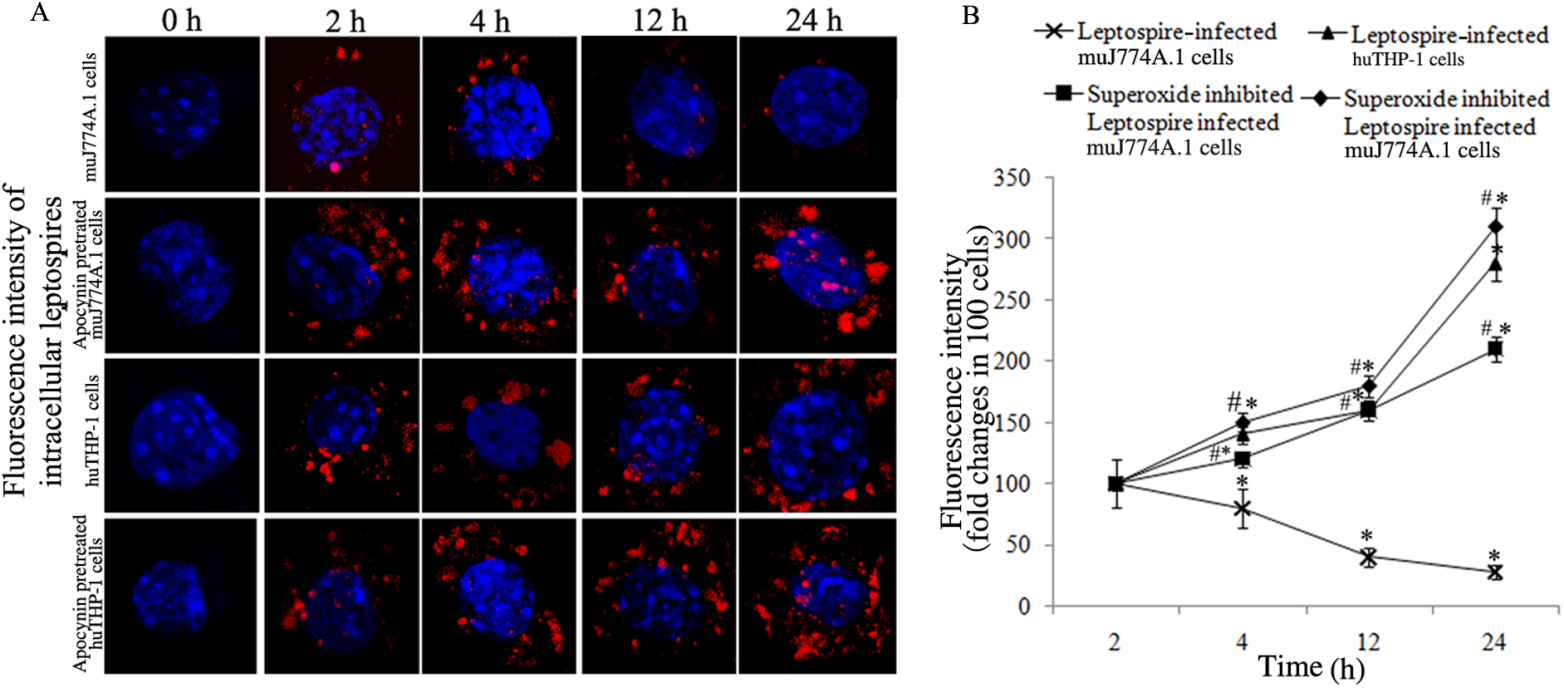
Fluorescence intensity of the *L*. *interrogans* in macrophages. J774A.1 and THP-1 cells were infected with *L*. *interrogans* strain Lai at an MOI of 100 for different time periods. The extracellular leptospires were killed by gentamicin, and the fluorescence intensity of intracellular leptospires were determined by confocal microscopy. Apocynin was used to inhibit the production of ROS. A. Leptospires in THP-1 or J774A.1 cells under a laser confocal microscope during infection with *L*. *interrogans* strain Lai for the indicated times. The blue plaques in the middle of the cells indicate the nucleus. The red spots around the nucleus indicate the intracellular leptospires. B. Statistical summary of the red fluorescence intensities that reflect leptospires in THP-1 or J774A.1 cells during infection with *L*. *interrogans* strain Lai for the indicated times. A minimum of 100 infected cells were measured in each experiment, and the data are expressed as the mean ± SD for three independent experiments. *P<0.05 versus 2 h of infection. ^#^*P* < 0.05 versus the cells without apocynin inhibition for the indicated times.

The leptospire-specific fluorescent staining assay confirmed that the relative fluorescence intensity levels rapidly increased in NADPH oxidase inhibitor apocynin pretreated J774A.1 cells, but continuously decreased in J774A.1 cells without apocynin treatment. Conversely, the relative fluorescence intensity levels gradually increased in both NADPH oxidase inhibitor apocynin pretreated THP-1 cells and THP-1 cells without apocynin pretreatment (P<0.05 Fig 6A and 6B). Moreover, less change was observed in the relative fluorescence intensity levels of leptospires in apocynin pretreated THP-1 cells and THP-1 cells without apocynin pretreatment.

### Viability of the *L*. *interrogans* strain Lai in different macrophages

**Fig 7:**
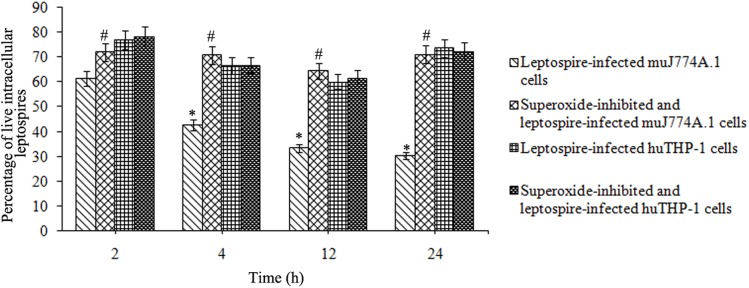
Viability of intracelluar *L*. *interrogans* in human and murine macrophages. J774A.1 and THP-1 cells were infected with *L*. *interrogans* strain Lai at an MOI of 100 for different time periods, the extracellular leptospires were killed by gentamicin, and the viability of intracellular leptospires was determined by staining with a Bacterial Viability Kit according to the manufacturer’s instructions followed by flow cytometry. Apocynin was used to inhibit the production of ROS. The results are expressed as the mean ± SD percentages of live leptospires for three independent experiments. *P<0.05 versus 2 h of infection. ^#^ *P*<0.05 versus cells without NADPH oxidase inhibitor apocynin pretreatment.

Viability assessment using a Bacterial Viability Kit confirmed that decreased percentages of live leptospires could be significantly rescued by the NADPH oxidase inhibitor apocynin in J774A.1 cells (P<0.05, Fig 7), while the percentages of live leptospires in J774A.1 cells without apocynin treatment dropped sharply (P<0.05, Fig 7). In addition, the percentages of live leptospires in THP-1 cells decreased slightly during the whole infection process compared to leptospires in EMJH medium (*P*<0.05), while less change was observed in the percentages of live leptospires in NADPH oxidase inhibitor apocynin pretreated THP-1 cells and THP-1 cells without apocynin pretreatment.

## Discussion

In individuals with no specific antimicrobial immunity, phagocytes play a critical role in eliminating the invading pathogens by phagocytosis. However, mononuclear macrophages, but not neutrophils, can kill the phagocytosed leptospires [11, 12, 33]. Therefore, the interaction between leptospires and mononuclear macrophages is an important occurrence during leptospirosis that decides the outcome of infection [12]. Our previous study noted that leptospires are capable of escaping host defense responses to maintain their own survival or replication in human macrophages but not in murine macrophages [13]. However, the mechanisms for these different intracellular fates of leptospires in macrophages of different hosts remains unclear [13]. Thus, an understanding of the strategies used by leptospires to evade host defenses or the mechanism by which macrophages of different hosts kill *L*. *interrogans* will enable a deep understanding of the pathogenic mechanism of leptospires.

During phagocytosis of microbial pathogens, phagocytes produce a high level of ROS to induce cellular oxidative stress and to promote the antimicrobial response [34]. ROS have been proven to contribute to murine resistance to *L*. *monocytogenes* infection and the listericidal activities of activated macrophages [35–37]. ROS levels of host cells can be measured with the ROS-specific fluorescent dye (DCFH-DA) [12, 19, 38]. In the present study, the ROS-specific fluorescent staining assay confirmed that infection with *L*. *interrogans* caused a rapid increase in ROS in J774A.1 and THP-1 cells, which confirmed the results reported by Hu W, et al [12] and Marangoni A [19], with more significant up-regulation of ROS in J774A.1 cells, and the increase could be significantly inhibited by the NADPH oxidase inhibitor apocynin. To further confirm the increase of ROS in leptospire infected-mouse and human macrophages, we used a superoxide assay kit to detect the superoxide levels [26, 27], and the results showed a rapid increase of superoxide in J774A.1 and THP-1 cells, with more significant up-regulation of superoxide in J774A.1 cells, and this increase could also be significantly inhibited by the NADPH oxidase inhibitor apocynin. The data indicated that *L*. *interrogans* infection caused NADPH-dependent ROS production in mouse and human macrophages, with more significant increases in ROS levels in mouse macrophages.

During a successful infection, pathogens such as *L*. *monocytogenes* perforate the membranous vacuole that contains them and escape into the macrophage cytoplasm, where they can grow, divide, and eventually nucleate host cell actin in a process that facilitates transfer to neighboring cells. However, if the macrophage is activated, localized reactive oxygen and nitrogen intermediates inhibit the escape of *L*. *monocytogenes* from vacuoles, and the bacteria are killed [16]. Our previous study demonstrated that leptospires resided in membrane-bound vacuoles in murine macrophages, but were free in the cytosol in human macrophages [13]; it is unknown whether these different intracellular distributions of *L*. *interrogans* are related to ROS production. Therefore, we used apocynin, an NADPH oxidase inhibitor [24, 25], to establish an inhibitory test as well as TEM to observe the effects of ROS on the intracellular distribution of leptospires in J774A.1 and THP-1 cells. The data from the present study showed that leptospires and remnants of leptospires within membrane-bound vacuoles in J774A.1 cells were significantly inhibited by apocynin, and leptospires outside of vesicular membranes were observed in the cytosol of apocynin pretreated J774A.1 cells. Moreover, the typical spiral shape and number of leptospires increased in a time-dependent manner in the cytosol of both apocynin pretreated J774A.1 and THP-1 cells. The results indicated that ROS contributed more to the retention of leptospires within vacuoles in activated mouse macrophages than in human macrophages, which resulted in free leptospires in the cytosol of human macrophages. It is similar to the report that ROI inhibit escape of *Listeria monocytogenes* from vacuoles in activated macrophages [16].

Most intracellular bacteria enter their host cells through an entry pathway in which phagocytotic vesicles are formed, and subsequently, the phagosomes fuse with lysosomes, and the pathogens are killed [39, 40]. In the present study, co-localization of intracellular *L*. *interrogans* and the late-endosomal/lysosomal marker LAMP-1 demonstrated that the co-localization of leptospires with lysosomes decreased significantly in apocynin pretreated J774A.1 and THP-1 cells. The co-localization data are consistent with the intracellular distribution of leptospires observed using TEM, which further confirmed the effect of ROS on the distinct intracellular location of the leptospires in mouse and human macrophages. The distinct intracellular fate of *L*. *interrogans* in THP-1 and J774A.1 cells, based on the determination results of intracellular distribution (TEM) and co-localization (Confocal Microscopy), is quite different from other pathogens such as *Francisella tularensis*, the fate of which is similar in THP-1 and J774A.1 cells (40).

To confirm that the ROS levels play a potential role in the different intracellular fates of *L*. *interrogans* in mouse and human macrophages that were demonstrated in a previous study [13], we used confocal microscopy to assess the amount of intracellular leptospires. The leptospire-specific fluorescent staining assay confirmed that the relative fluorescence intensity levels of intracellular leptospires increased in apocynin pretreated J774A.1 and THP-1 cells after 2 h of infection. Furthermore, to confirm the viability of intracellular leptospires, we used a Bacterial Viability Kit to determine the live or dead leptospires in the macrophage [13, 32]. The results showed that clearly that most of the leptospires in J774A.1 cells are killed in 24 h whereas in human macrophages they continue to grow, which is consistent with our previous study [13]. The viability of leptospires in THP-1 cells is also similar to the viability changes of intracellular *Coxiella burnetii* in THP-1 cell determined with the same methods reported by Ghigo E, *et al*. [31]. The decreased viability of leptospires in J774A.1 cells could be rescued in apocynin pretreated J774A.1 cells and that less change occurred in apocynin pretreated THP-1 cells compared with THP-1 cells without apocynin pretreatment (Fig 7). The viability of intracellular leptospires has confirmed the changes of intratracellular leptospires shown in Fig 6. Changes in the amount and viability of intracellular leptospires were also consistent with the results from TEM, which suggested that ROS contributed to the killing of leptospires in mouse and human macrophages, but this contribution was less in human macrophages.

## Conclusions

In the present study, we demonstrated that infection with *L*. *interrogans* caused significant increases in ROS in mouse macrophages but lower increases in human macrophages.

ROS contributed substantially to the bactericidal ability of mouse macrophages to kill intracellular leptospires but contributed to a lesser extent in human macrophages, which partially explains the different intracellular fates of *L*. *interrogans* in human and mouse macrophages as well as the differences in the severity of leptospirosis in humans versus reservoir maintenance hosts.
